# Effect of Cu Film Thickness on Cu Bonding Quality and Bonding Mechanism

**DOI:** 10.3390/ma17092150

**Published:** 2024-05-04

**Authors:** Tsan-Feng Lu, Kai-Ning Hsu, Ching-Chi Hsu, Chia-Yu Hsu, YewChung Sermon Wu

**Affiliations:** Department of Materials Science and Engineering, National Yang Ming Chiao Tung University, Hsinchu 30010, Taiwan; s0881513.c@nycu.edu.tw (T.-F.L.); kaining0927.en10@nycu.edu.tw (K.-N.H.); peter0429.en09@nycu.edu.tw (C.-C.H.); chiayu.ii12@nycu.edu.tw (C.-Y.H.)

**Keywords:** Cu–Cu direct bonding, surface creep, elastic deformation, void morphology, Cu film thickness

## Abstract

In the hybrid bonding process, the final stage of chemical mechanical polishing plays a critical role. It is essential to ensure that the copper surface is recessed slightly from the oxide surface. However, this recess can lead to the occurrence of interfacial voids between the bonded copper interfaces. To examine the effects of copper film thickness on bonding quality and bonding mechanisms in this study, artificial voids were intentionally introduced at the bonded interfaces at temperatures of 250 °C and 300 °C. The results revealed that as the thickness of the copper film increases, there is an increase in the bonding fraction and a decrease in the void fraction. The variations in void height with different copper film thicknesses were influenced by the bonding mechanism and bonding fraction.

## 1. Introduction

For decades, transistors have been consistently miniaturized to comply with Moore’s Law. One of the promising candidates for extending Moore’s Law is three-dimensional integrated circuits (3DICs). 3DIC interconnection has emerged as an advanced packaging technology and has been extensively researched [1,2,3,4,5]. In the past, with the miniaturization of solder joints, various reliability issues have arisen, such as side wetting and bridge failure [6,7]. To address these concerns, the hybrid bonding technique was proposed [8,9,10].

Hybrid bonding techniques, which combine Cu bonding and oxide bonding, have emerged as important methods for 3DIC applications [8,9,10,11]. In the hybrid bonding process, the final chemical mechanical polishing (CMP) stage is crucial for achieving a successful bond. During this stage, it is essential to achieve a flat oxide surface while ensuring that the Cu is slightly recessed from the oxide surface. The degree of “Cu recess” depends on several factors, including temperature and Cu film thickness. However, interfacial voids can occur between bonded Cu interfaces due to this recess. This may lead to poor connections or even electrical discontinuity.

Through-silicon vias (TSVs) play a crucial role in the packaging of 3DICs. In addition to TSVs, hybrid bonding techniques that involve both Cu bonding and oxide bonding have also been developed for 3DIC applications. Typically, oxide–oxide bonding occurs at room temperature before the temperature is raised to around 300 °C for Cu–Cu bonding [12,13,14,15,16]. The requirement to elevate the temperature is attributed to the higher coefficient of thermal expansion (CTE) of Cu compared to the surrounding SiO_2_ [17]. At elevated temperatures, the Cu pad protrudes, establishing contact with the Cu pad on the opposite side, thus forming a permanent interconnection.

The success of the hybrid bonding process relies on the final chemical mechanical polishing (CMP) stage of the copper [18,19]. During this stage, it is essential to achieve a flat oxide surface while ensuring that the copper is slightly recessed from the oxide surface [12,13,14]. The degree of “Cu recess” required depends on several factors, including the bonding temperature [20,21], expected service temperature, and the depth of TSV (Cu film thickness). Although achieving the correct Cu recess is crucial, interfacial voids can arise between bonded Cu interfaces because of this recess [22,23,24].

In this study, artificial voids were introduced at bonded interfaces to investigate the effect of Cu film thickness on the bonding quality and bonding mechanism at temperatures of 250 °C and 300 °C.

## 2. Experimental

### 2.1. Cu Film Electrodeposition

Electroplated Cu film on Si wafer was used in this study. A 50 nm of Ta was sputtered onto a Si wafer as the adhesion layer, followed by the sputtering of a 200 nm thick Cu seed layer. The Si substrate was then immersed in the electrolyte during electroplating. After the electroplating process, the surface of the Cu film was flattened using chemical mechanical polishing (CMP).

Three different thicknesses of Cu films were used in the study: 0.8 μm for sample 1, 2.0 μm for sample 2, and 2.7 μm for sample 3. These different thicknesses allowed for the investigation of the influence of Cu film thickness on the bonding quality and bonding mechanism.

### 2.2. Pre-Treatment of the Specimens

Wafers were then diced into 1 × 1 cm^2^ pieces. To investigate the evolution of voids, artificial voids were formed on a flat unetched surface (denoted as F surface) and a wet-etched surface (W surface). The sample fabrication processes are described in detail in reference [22,23].

### 2.3. Material Characterizations

**Figure 1:**
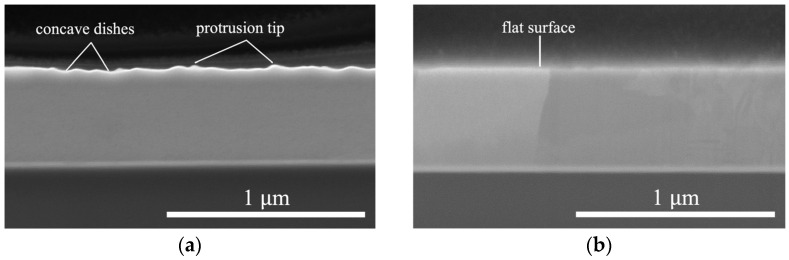
Cross-sectional SEM images of the (**a**) W and (**b**) F surfaces.

**Figure 2:**
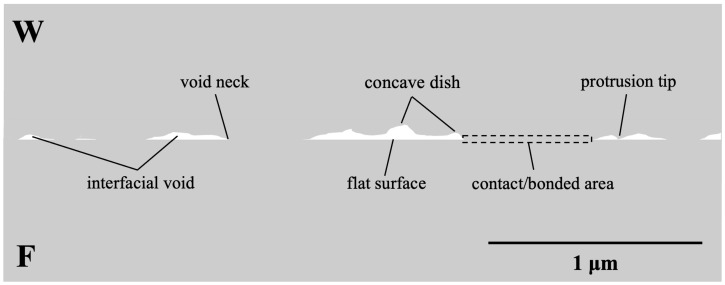
A schematic illustration of the contacted interface of the W and F surfaces.

Prior to bonding, the surface roughness of the Cu film (over a 10 × 10 µm^2^ area) was measured using atomic force microscopy (AFM, Bruker Dimension Icon Scanning Probe Microscope (ICON)) (Bruker, Hsinchu, Taiwan). The root mean square (*R*_q_) roughness of the F surface of sample 1 was measured to be 1.53 nm, while that of the W surface was 14.8 nm. Cross-sectional scanning electron microscopy (SEM) images of sample 1, measured by using focused ion beam (FIB, Helios NanoLab 650) (FEI, Hillsboro, Oregon, United States) techniques, revealed that the F surface was very flat, while the W surface had protrusion tips and concave dishes as shown in Figure 1. The samples were mated together at room temperature. A schematic illustration of the bonded interface, based on the cross-sectional SEM images of the W and F surfaces, is shown in Figure 2.

After the bonding and annealing processes, the samples were subjected to grinding and polishing. Cross-sectional specimens for SEM analysis were then prepared using FIB techniques. This allowed for detailed observation and characterization of the voids and interfaces in the samples.

### 2.4. Bonding Process

The samples were arranged in a differential thermal expansion fixture that was composed of stainless steel and aluminum. This fixture was identical to the one proposed in our previous work [25]. Initially, at room temperature, a minimal compressive load was applied to the sample stack. As the processing temperature increased, the compressive stress on the sample stack increased due to the differential thermal expansion between the various materials. Assuming uniform distribution of stress on the 1 × 1 cm^2^ sample, the calculated uniform compressive stress, σ, was 53.64 MPa at 250 °C and 65.56 MPa at 300 °C. However, it is important to note that the actual stress experienced by the sample could not be accurately determined due to the occurrence of creep deformation in the Cu films at elevated temperatures. Further details regarding the deformation are discussed in depth in the section dedicated to Cu bonding mechanism.

Artificial voids were formed at the bonded interfaces by bonding the samples for 0.5 h at 250 °C (referred to as B250t0.5) and 300 °C (B300t0.5). The bonding process took place in an ordinary vacuum of 1.33 × 10^−1^ Pa. To observe the evolution of the voids, subsequent vacuum annealing was performed at the same bonding temperature for an additional 0.5 h, resulting in samples denoted as B250t1 and B300t1.

## 3. Results and Discussion

**Figure 3:**
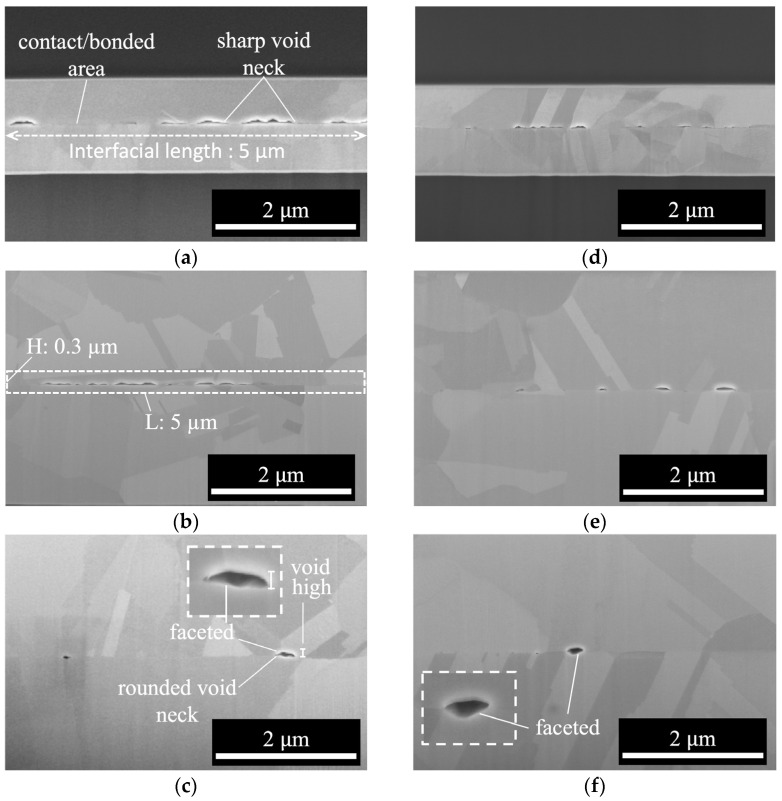
Cross-sectional SEM images of samples bonded at 250 °C: (**a**) 1B250t0.5, (**b**) 2B250t0.5, (**c**) 3B250t0.5, (**d**) 1B250t1, (**e**) 2B250t1, and (**f**) 3B250t1.

Figure 3 presents cross-sectional SEM images of B250, illustrating the morphologies of the voids. These images were utilized to evaluate the bonding quality using metrics such as bonding fraction (BF), void fraction (VF), and void height (VH). BF is determined by estimating the bonded length (projection of bonded/contact areas on the image) versus the “interfacial length” (5 µm), as illustrated in Figure 3a. VF is determined by comparing the void areas to the areas around the interface (0.3 × 5 µm^2^), while VH represents the height of voids, as depicted in Figure 3b,c. By analyzing the BF, VF, and VH, the bonding quality and mechanism of the samples were evaluated.

Table 1 presents the measured values of BF, VF, and VH for the bonded interfaces of the samples bonded at 250 °C (B250). It reveals a notable increase in BF with the increasing thickness of the Cu film. The BF of 3B250t0.5 was 93.06%, and higher than that of 2B250t0.5 (61.14%) and 1B250t0.5 (31.24%). Similarly, the BF of 3B250t1 was 95.10%, and greater than that of 2B250t1 (86.66%) and 1B250t1 (69.75%). The primary reason for the increase in BF can be attributed to the greater creep/elastic deformation of the Cu film as its thickness increases. This will be further discussed in the following paragraphs.

Two mechanisms, diffusion and deformation by yielding or creep, were employed to describe the morphology of voids in Cu bonding [26]. The related deformed morphologies have been simplified as voids closed by deformation to have sharp necks and voids closed by diffusion flow to have rounded necks.

In our previous studies, we extensively investigated these two mechanisms, specifically focusing on the morphologies of void surfaces [22,23,24]. The creep deformation mechanism is primarily driven by a high stress concentration and stress gradient, while the diffusion mechanism is influenced by a reduction in surface free energy. In the case of creep deformation, Cu atoms diffuse from the high compressive stress regions (around contact areas) towards stress-free and tensile stress regions (such as the neck, dish, and flat regions) in order to relieve stress [22,23,24].

In a relevant study on bonding in (111)-oriented nano-twinned Cu, Juang et al. [27] employed a diffusion creep mechanism. Their experiment assumed an average distance, *l*, between the center of a contacted/bonded region and an un-contacted/void region, which led to the determination of the creep rate.

**Figure 4:**
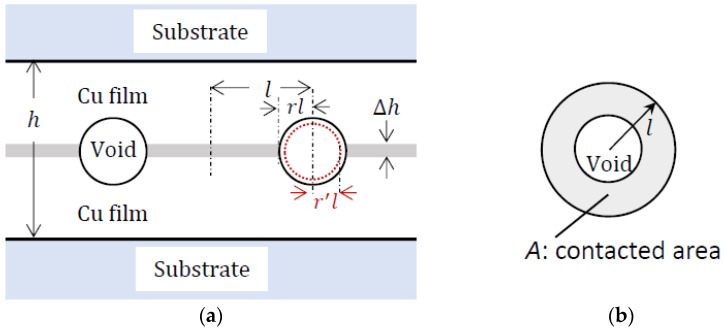
Schematic diagrams of the (**a**) cross-section and (**b**) top view of the part of the bonded interface.

In this study, a similar approach was adopted to investigate the influence of Cu film thickness on the bonding quality and bonding mechanism. As depicted in Figure 4, for the purpose of analysis and calculation simplification (reducing variables), it is assumed that the void shape is spherical with an average radius of *rl* (where *r* < 1). Based on this assumption, the bonding fraction can be estimated as follows:

(1) $$\mathrm{BF}\left(\%\right)=\frac{2l-2rl}{2l}\times 100=\left(1-r\right)\times 100$$

The total thickness of the Cu film is denoted as *h*, and Δ*h* represents the change in thin film thickness under compression. The elastic strain, *ε*, can be calculated as follows:

(2) $$\varepsilon =\frac{\Delta h}{h}=\frac{\sigma }{Y},$$

where *σ* represents the uniform compressive stress at the contacted area and *Y* is the Young’s modulus of Cu. As mentioned earlier, the calculated compressive stress for the samples at 250 °C was determined to be 53.64 MPa, indicating that the applied compressive stress (*σ*) was the same for all three samples. This implies that the strain (*ε*) experienced by the samples was also the same.

The change in thin film thickness, Δ*h*, can be inferred to be directly proportional to the initial thickness, *h*. The initial length of *h*_3_ was 5.4 μm, which was 1.35 times greater than *h*_2_ (4.0 μm) and 3.38 times greater than *h*_1_ (1.6 μm). Therefore, Δ*h*_3_ would be approximately 1.35 times greater than Δ*h*_2_ and 3.38 times greater than Δ*h*_1_.

To study the effect of Cu thickness on the bonding fraction (BF), two volumes were considered: the strained volume (*V_strained_*) and the reduced void volume (Δ*V_void_*). The objective of creep deformation is to relocate all the atoms within the strained volume (*V_strained_*) from the bonded region to the void region [27]. The strained volume can be estimated as follows:

(3) $$V_{strained}=A\Delta h,$$

where *A* represents the contacted area given as follows:

(4) $$A={[l}^{2}-{\left(rl\right)}^{2}]\pi .$$

Thus, the strained volume can be expressed as follows:

(5) $$V_{strained}=[1-r^{2}]\pi l^{2}\Delta h.$$

On the other hand, the reduced void volume (∆*V_void_*) can be determined by considering the change in void radius after creep deformation. It can be calculated as follows:

(6) $${∆V}_{void}=\frac{4\pi \left[{\left(lr\right)}^{3}-{\left(lr^{\prime }\right)}^{3}\right]}{3},$$

where *lr′* represents the new void radius after the creep deformation. When we equate the two expressions of volume, we obtain the following:

(7) $$\left[1-r^{2}\right]\pi l^{2}\Delta h=\frac{4\pi \left[{\left(lr\right)}^{3}-{\left(lr^{\prime }\right)}^{3}\right]}{3},$$

(8) $${\left(r^{\prime }\right)}^{3}=r^{3}-\frac{3\left[1-r^{2}\right]\Delta h}{4l}$$

This indicates that as Δ*h* increases, *r′* decreases. As mentioned before,

(9) $$\mathrm{BF}=1-r^{\prime },$$

which means that BF increases as *r′* decreases. Therefore, BF increased with the increase in Δ*h*.

Since Δ*h*_3_ was greater than Δ*h*_2_ and Δ*h*_1_, the bonding fraction (BF) of 3B250t0.5 was greater than that of 2B250t0.5 and 1B250t0.5. Similarly, the BF of 3B250t1 was greater than that of 2B250t1 and 1B250t1.

**Figure 5:**
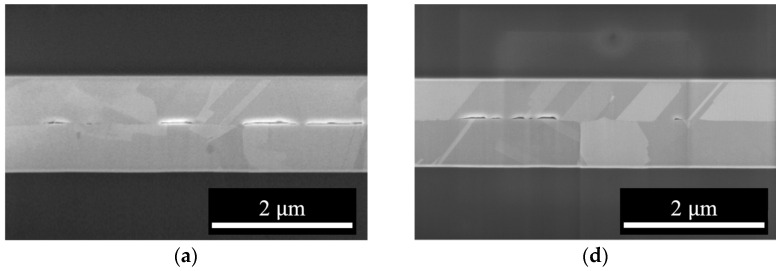

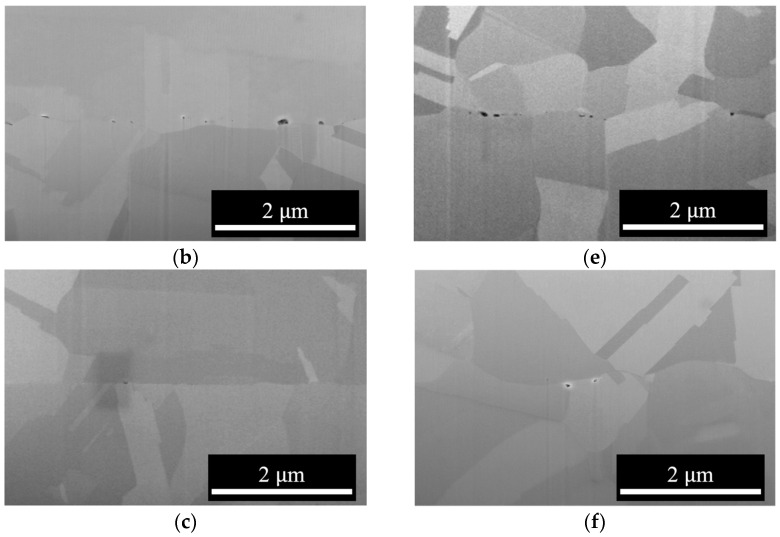
SEM cross-sectional images of samples bonded at 300 °C: (**a**) 1B300t0.5, (**b**) 2B300t0.5, (**c**) 3B300t0.5, (**d**) 1B300t1, (**e**) 2B300t1, and (**f**) 3B300t1.

It can be concluded that the same trend applies to samples bonded at 300 °C (B300), as seen in Table 2 and Figure 5. The BF of the 3B300t0.5 sample was 98.62%, which was higher than that of 2B300t0.5 (88.79%) and 1B300t0.5 (54.30%). Similarly, the BF of the 3B300t1 sample was 95.58%, which was greater than that of the 2B300t1 (90.70%) and 1B300t1 (77.43%) samples.

Table 1 also indicates that the VF decreased as the thickness of the Cu film increased. For instance, the VF of the 3B250t0.5 sample was 0.75%, which was lower than that of 2B250t0.5 (1.27%) and 1B250t0.5 (2.70%). Similarly, the VF of the 3B250t1 sample was 0.93%, which was lower than that of 2B250t1 (0.95%) and 1B250t1 (1.49%). This observation remains valid for samples bonded at 300 °C (B300), as evidenced by the data presented in Table 2. The decrease in VF can be explained by the corresponding increase in both creep deformation and BF as the thickness of the Cu film increases.

As previously mentioned, VF was determined by calculating the ratio of the void areas against the areas around the interface. According to Figure 4, the estimate of VF is adjusted using the following equation:

(10) $$\mathrm{VF}=\pi {\left(lr^{\prime }\right)}^{2}/2lH.$$

Here, *H* represents the height of the areas around the interface, as shown in Figure 3b. It can be observed that VF increases with (*r′*)^2^, which in turn increases with a decrease in Δ*h*. Hence, as Δ*h* decreases, the VF increases. Consequently, sample 1 has a greater VF compared to samples 2 and 3.

The changes in VH with varying Cu film thickness were influenced by the bonding mechanism. This will be discussed in the following paragraphs.

As mentioned earlier, the diffusion mechanism occurs through a reduction in surface free energy. As illustrated in Figure 2, there are four types of free energies associated with the morphologies of void surfaces: free energy at the protrusion tip (*G_+tip_*), free energy at the flat surface (*G_flat_*), free energy at the concave dishing (*G_−dish_*), and free energy at the void neck (*G_−neck_*). The diffusion of Cu atoms from protrusion tips to flat surfaces, dishing regions, and void necks reduces the free energy, resulting in an increase in BF and VH [23].

Additionally, as Cu atoms diffuse from the flat surface (F surface) towards the void necks, some F surfaces undergo a change in their radius of curvature (*R_flat_*) from infinite to negative or faceted, leading to the formation of lenticular and faced voids, as shown in Figure 3c,f. These findings are in agreement with those of Gondcharton et al. [28], who studied Cu–Cu bonded structures. This “F surface” diffusion also contributes to an increase in VH.

As shown in Figure 3a,b, the relatively sharp morphologies of the void necks in 1B250t0.5 and 2B250t0.5 suggest that the evolution of voids in these samples was primarily influenced by creep deformation. In contrast, the rounded neck and lenticular shape of the 3B250t0.5 voids (Figure 3c) suggests a different mechanism, namely diffusion.

Considering that the bonding mechanism of both 1B250t0.5 and 2B250t0.5 was dominated by deformation and taking into account that the VF (void fraction) of 2B250t0.5 was less than that of 1B250t0.5, it can be inferred that the VH (void height) of 2B250t0.5 was less than that of 1B250t0.5.

However, in the case of 3B250t0.5, the VH was greater than that of 2B250t0.5, despite the VF of 3B250t0.5 being less than that of 2B250t0.5. This discrepancy can be attributed to the fact that the bonding mechanism of 3B250t0.5 was primarily dominated by diffusion, which led to an increase in the VH.

Therefore, it can be concluded that the morphology and evolution of voids in the bonded samples are influenced by both the bonding mechanism (deformation or diffusion) and the VF, resulting in variations in the VH among the different samples.

This transition in the dominant bonding mechanism can be attributed to the increase in bonding fraction (BF). This change can be understood by considering the atomic creep flux, as elucidated by Juang et al. [27] in their study on the bonding of nano-twinned Cu [29].

The flux can be represented by the following equation:

(11) $$J=D\sigma_{contact}/kTl,$$

where *J* is the creep flux, *D* is the diffusivity of Cu, *σ_contact_* is the actual stress at the contact area, *k* is Boltzmann’s constant, *T* is the absolute temperature, and *l* represents the average distance between the center of a bonded region and a void region (as illustrated in Figure 2).

The stress at the contacted area can be estimated as follows:

(12) $$\sigma_{contact}\approx \;C\sigma /BF,$$

where *C* is a proportionality constant and *σ* is the uniform compressive stress as mentioned earlier. This relationship indicates that as the bonding fraction (BF) increases, the actual stress at the contacted area (*σ_contact_*) decreases. This implies that the influence of creep deformation (creep flux) decreases with an increase in BF. When the bonding fraction is high, the contribution of creep deformation to the overall bonding mechanism diminishes, and the dominant mechanism transitions to diffusion.

As previously mentioned, the increase in Cu film thickness is associated with a rise in BF, indicating that the bonding mechanism of thicker Cu is more likely to be dominated by diffusion. Concurrently, VH also increases when the bonding mechanism is predominantly governed by diffusion.

This conclusion applies equally to the different groups of specimens B250t1, B300t0.5, and B300t1. Regardless of the specific group, when the bonding fraction (BF) increases, the influence of creep deformation gradually weakens, and the diffusion mechanism becomes the dominant bonding mechanism. Based on observations of Figure 3 and Figure 5, it is noted that in specimens 2B250t1, 3B250t1, 2B300t0.5, 2B300t1, 2B300t1, and 3B300t1, the dominant bonding mechanism is diffusion.

## 4. Conclusions

Artificial voids were introduced at bonded interfaces to investigate the influence of Cu film thickness (the depth of TSV) on bonding quality and bonding mechanisms at temperatures of 250 °C and 300 °C.

Three different thicknesses of Cu films were utilized: (1) 0.8 μm, (2) 2.0 μm, and (3) 2.7 μm. The findings indicate an increase in the bonding fraction (BF) and a decrease in the void fraction (VF) as the thickness of Cu film increases. This can primarily be attributed to the greater creep/elastic deformation exhibited by the Cu film as its thickness increases.

The variations in void height (VH) with different Cu film thicknesses were influenced by the bonding mechanism and BF. In general, deformation mechanisms lead to a decrease in VH, while diffusion results in an increase. The bonding mechanism of thicker Cu is more likely to be dominated by diffusion, as evidenced by the increase in BF with increasing Cu film thickness. This dominance of diffusion is attributed to the decrease in actual stress at the contacted area (*σ_contact_*) and the reduced influence of creep deformation (creep flux). These experimental findings are supported by the surface diffusion creep model.

To sum up, our study confirms through experiments and modeling that increasing the thickness of the Cu thin film is beneficial for Cu–Cu bonding. As bonding temperature and Cu joint size decrease, Cu thermal expansion also decreases [30,31,32]. For a high-quality Cu–Cu bonding interface, it is crucial to consider the depth of the Cu joints. In the future, we intend to further validate these findings using hybrid bonding, which we believe will be advantageous for 3D package applications.

## Figures and Tables

**Table 1:** The measured bonding fraction (BF), void fraction (VF), and void height (VH) of the samples bonded at 250 °C (B250).

	1B250t0.5	2B250t0.5	3B250t0.5	1B250t1	2B250t1	3B250t1
BF (%)	31.24	61.14	93.06	69.75	86.66	95.10
VF (%)	2.70	1.27	0.75	1.49	0.95	0.93
VH (nm)	38.1–101.5	18.1–60.3	38.7–73.0	19.0–36.5	50.8–69.8	9.5–136.4

**Table 2:** The measured bonding fraction (BF), void fraction (VF), and void height (VH) of samples bonded at 300 °C (B300).

	1B300t0.5	2B300t0.5	3B300t0.5	1B300t1	2B300t1	3B300t1
BF (%)	54.30	88.79	98.62	77.43	90.70	95.58
VF (%)	1.96	0.72	0.08	1.01	0.56	0.13
VH (nm)	50.8–66.6	31.7–44.4	22.2	41.2–53.9	47.6–84.6	19.0–44.4

## Data Availability

The data supporting the findings of this study are available from the corresponding author upon reasonable request.
